# Low Temperature Joining and High Temperature Application of Segmented Half Heusler/Skutterudite Thermoelectric Joints

**DOI:** 10.3390/ma13010155

**Published:** 2019-12-31

**Authors:** Weian Wang, Xiaoya Li, Ming Gu, Yunfei Xing, Yefeng Bao

**Affiliations:** 1 The State Key Lab of High Performance Ceramics and Superfine Microstructure, Shanghai Institute of Ceramics, Chinese Academy of Sciences, Shanghai 200050, China; weianwangwwa@163.com (W.W.); ftgm@mail.sic.ac.cn (M.G.); yunfeix@student.sic.ac.cn (Y.X.); 2 School of Mechanical and Electrical Engineering, Hohai University, Changzhou 213022, China; baoyf@hhuc.edu.cn

**Keywords:** segmented thermoelectric joints, joining process, microstructure, contact resistance

## Abstract

A low temperature joining process has been developed to fabricate segmented half Heusler/skutterudite thermoelectric joints, and high temperature service behavior of the joints has been studied. The microstructure and electrical resistance across the joint before and after aging were investigated. The joint is well bonded and no cracks appear at the interfaces of the joint before and after aging, which can attribute to the formation of high melting point intermetallic compounds. The electrical resistance crosses the bonding layer smoothly and the contact resistance is low. These results show the process is effective, and promising for preparation of segmented thermoelectric devices.

## 1. Introduction

Thermoelectric (TE) materials that can convert heat into electricity directly have found important applications in radio-isotope thermoelectric generators used in space exploration [1], and also become increasingly appealing for waste heat recovery in industries [2]. The energy conversion efficiency of TE materials depends not only on the temperature difference between the hot and cold ends and the hot side temperature, but also on the dimensionless figure of merit (ZT) of the materials used, ZT = *α*^2^*σT*/*κ*, where *α* is the Seebeck coefficient, *σ* is the electrical conductivity, *T* is the absolute temperature, and *κ* is the thermal conductivity. For given dimensions (length and cross-sectional area), hot side temperature and temperature difference between hot and cold ends, higher ZT TE materials—such as Bi_2_Te_3_ [3,4,5], ZnSb [6,7], PbTe [8,9], CoSb_3_ [10,11], half-Heusler [12,13], etc.—can give higher conversion efficiency. However, the reported conversion efficiency rarely exceeds 10% because no single TE material has high ZT over a wide temperature range, and each TE material possesses high ZT within a certain temperature range [14]. Therefore, it is necessary to use different TE materials in each temperature range to get high ZT and conversion efficiency over a wide temperature range. Ouyang [15] reported a calculated conversion efficiency up to 20.9% over a temperature range of 300 K to 1000 K for segmented devices made of state-of-the-art TE materials. Kang [16] demonstrated a conversion efficiency of 17% for a segmented element made of SiGe, PbTe, and Bi_2_Te_3_ based alloys by mechanical contact.

The process for fabricating segmented TE devices is complicated and challenging, which deals with bulk TE material preparation, barrier layer design and fabrication, TE materials metallization, different TE materials joining in series, dicing, and p- and n- type TE materials connecting to electrode. Among them the most difficult is to join different TE materials in series. Li [17] joined n-type Bi_2_Te_3_/PbSe_0.5_Te_0.5_ and p-type Bi_0.3_Sb_1.7_Te_3_/Zn_4_Sb_3_ by one step spark plasma sintering. Wannasut [18] joined YBa_2_Cu_3_O_7−x_/Na_y_CoO_2_ by conventional sintering. Zhang [19] joined n-type Bi_2_Te_2.97_Se_0.3_/Yb_0.3_Co_4_Sb_12_ and p-type Bi_0.48_Sb_1.52_Te_3_/CeFe_4_Sb_12_ by Sn soldering. Hung [20] joined Ca_2.8_Lu_0.15_Ag_0.05_Co_4_O_9+δ_/Ti_0.3_Zr_0.35_Hf_0.35_CoSb_0.8_Sn_0.2_ by Ag brazing. When sintering is adopted to produce segmented TE materials, the sintering temperature should be adjusted to avoid insufficient or overheating of the sintered thermoelectric materials, which may affect the TE properties of the materials. Bulat [21] tried to solve this problem by developing temperature gradient sintering technique. Brazing is another choice, but usually the brazing temperature is much higher than the service temperature of the low temperature segment. Therefore, brazing may affect the TE properties of the material. Besides, Sn soldering—a low temperature joining method that has no bad effect on the TE properties of the materials—is also worth trying. The problem with Sn soldering is that the soldered joints will become porous and even get cracking after long time service at high temperatures [22], which may increase the electrical resistance of the joint. In this paper we report a new process for low temperature joining of segmented thermoelectric materials, which succeeded in avoiding the bad effect on the TE properties of the low temperature segments. Also reported is the high temperature service behavior of the segmented TE joints.

## 2. Materials and Methods

The process includes four steps: fabrication of bulk TE materials with barrier/connecting layer, preparation of joining material including low temperature and high temperature components, connection of the bulk TE materials with the joining material under suitable temperature and pressure, and heat treatment of the joint to turn the joining material into intermetallic compounds (IMCs) to withstand high temperature. An n-type segmented skutterudite (SKD, Yb_0.3_Co_4_Sb_12_) and half Heusler (HH, Hf_0.5_Zr_0.5_NiSn) TE joint was fabricated by this process. Bulk SKD with Ni and Ti-Al alloy layers was fabricated according to references [23,24,25]. Ni served as connecting layer, and Ti-Al as barrier layer. Bulk HH was prepared according to reference [13]. Ni was electroplated on HH as a connecting layer. 300-mesh Ni (40 wt%) and Sn (60 wt%) powders were mixed as joining material. The HH and SKD segments were connected as a HH/Ni/Ni-Sn/Ni/Ti-Al/SKD joint under vacuum at a temperature of 280 °C and a pressure of 5 MPa for 5 min. The resulting joint was then heat treated at 340 °C for 30 min.

In order to demonstrate that the joint can withstand high temperature service, an accelerated isothermal aging experiment was carried out on samples with dimensions of 4 × 4 × 6 mm^3^ cut from the joint by electrical discharge machine. Since the optimal practical application temperature of SKD is up to 550 °C, the isothermal aging temperature was set as 600 °C. The aging lasted for 168 h.

The microstructure across the joint interfaces before and after aging was characterized by scanning electron microscopy (SEM, ZEISS, SUPRA-55-SAPPHIRE, Oberkochen, Germany) and energy dispersive spectroscopy (EDS, OXFORD, X-MaxN, Oxfordshire, UK). The electrical resistance across the joint before and after aging and the contact resistivity at interfaces were measured on a homemade four-probe platform, and the method was detailed in reference [26].

## 3. Results and Discussion

**Figure 1:**
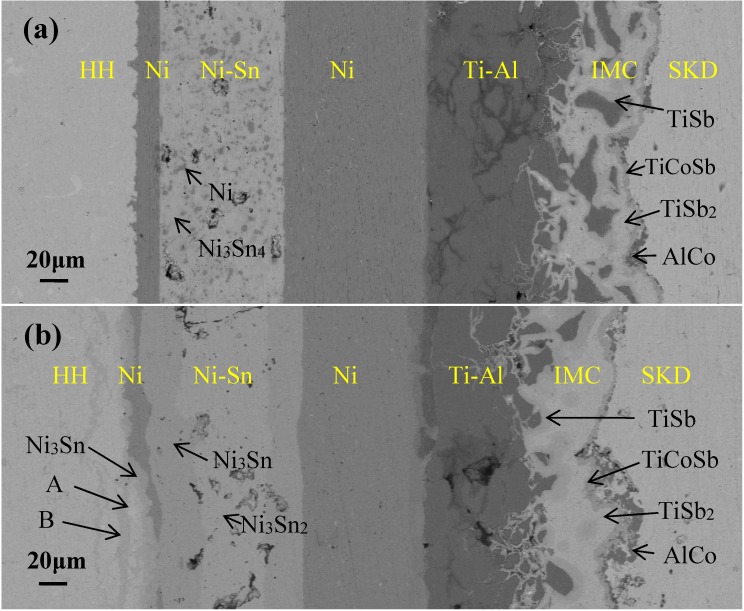
SEM (scanning electron microscopy) image of the microstructure across the joint interfaces before (**a**) and after (**b**) isothermal aging at 600 °C for 168 h. A and B denote Hf and Zr substituted Ni_3_Sn, and Ni riched HH, respectively.

Figure 1 shows the microstructure across the joint interfaces before and after aging. As can be seen, the joint is well bonded, and no cracks appear at each interface of the HH/Ni/Ni-Sn/Ni/Ti-Al/SKD joint except some voids in Ni-Sn bonding layer (Figure 1a). The bonding layer that forms good bonding with the connecting layer (Ni) on both sides of SKD and HH consists mainly of light grey substrate and dark grey phase, which were identified by EDS as Ni_3_Sn_4_ and residual Ni particles. During the joining process Sn powder melted and reacted with Ni, resulting in metallurgical bonding of the joint with high strength [27]. In the subsequent heat treatment, Sn continued to react with Ni and became Ni_3_Sn_4_ until Sn was consumed entirely. As indicated in the Ni-Sn phase diagram [28], the melting temperature of Ni_3_Sn_4_ is 794.5 °C. Therefore, the Ni-Sn bonding layer should have high heat resistance temperature. Sn transforms into Ni_3_Sn_4_ causes shrinkage [29], which may be reason why voids appear in the bonding layer. Although Ni was electroplated on HH, it was firmly bonded because the rough surface of HH enables Ni to clamp HH, which leads to mosaic effect and increase of bonding strength. At the Ti-Al/SKD interface, there is an IMCs layer, EDS shows that they are AlCo, TiCoSb, TiSb, and TiSb_2_ phases formed during SPS sintering according to Gu [24] and Tang [25].

After the isothermal aging, the joint is still well bonded (Figure 1b). However, the microstructure across the joint changed. The residual Ni particles in the bonding layer disappeared completely, and the connecting layer (Ni) on both sides of SKD and HH became much thinner than that before isothermal aging. EDS investigation was conducted on the bonding layer. The results show that the atomic percentage of the dark grey area is about 74.9% at% Ni and 25.1% at% Sn, and that of light grey area is 60 at% Ni and 40 at% Sn, which can be assumed as Ni_3_Sn and Ni_3_Sn_2_, respectively, according to the Ni-Sn phase diagram [28]. In the process of isothermal aging, the Ni and Sn atoms in the bonding layer and at the interfaces underwent solid state diffusion and reaction and transformed into Ni_3_Sn_2_ and Ni_3_Sn. The melting temperature of Ni_3_Sn_2_ and Ni_3_Sn IMCs is 1280 °C and 1189 °C [28], respectively. So the Ni-Sn bonding layer has high heat resistance temperature, which may be the reason why the joint could withstand the isothermal aging experiment and remain well bonded. At the HH/Ni interface of the joint, the microstructure became very complicated after isothermal aging because of the solid state diffusion and reaction between the connecting layer (Ni) and HH. EDS investigation results show, from Ni to HH, they are residual Ni, Ni_3_Sn (75.7 at% Ni, 24.3 at% Sn), Hf and Zr substituted Ni_3_Sn (A: 72.0 at% Ni, 23.0 at% Sn, 2.6 at% Hf and 2.4 at% Zr), Ni enriched HH (B: 68.1 at% Ni, 16.0 at% Sn, 8.3 at% Hf and 7.6 at% Zr) and HH (33.4 at% Ni, 33.1 at% Sn, 17.3 at% Hf and 16.2 at% Zr). At the Ti-Al/SKD interface the amount of IMCs increased significantly after aging. Gu [24] and Tang [25] have analyzed in detail the microstructure evolution and phase composition of SKD/Ti-Al/Ni after thermal aging, which is not explored here.

**Figure 2:**
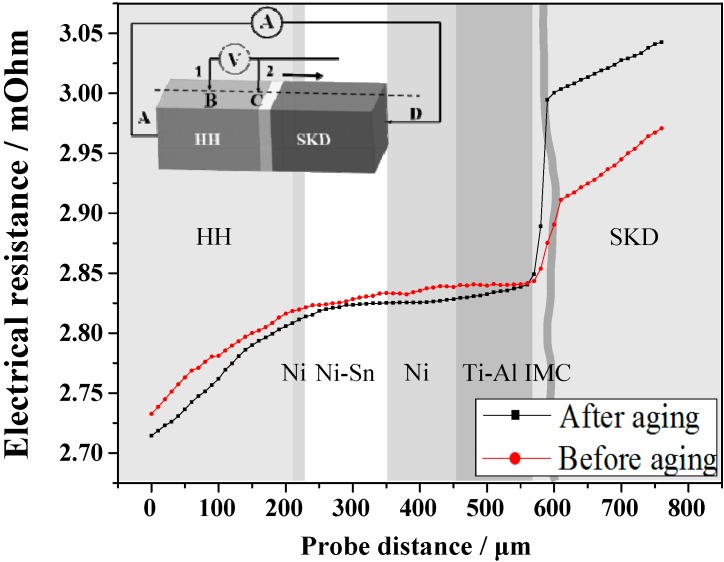
Electrical resistance across the HH/SKD joint before and after isothermal aging.

The effectiveness of a method for joining segmented TE materials depends not only on good bonding of the joint but also on low contact electrical resistance at the interfaces, because the electrical resistance across the joint has great effect on the performance of the segmented TE materials [30]. Smaller electrical resistance across the joint produces less Joule heat, which can reduce the loss of the performance of the segmented TE materials. The electrical resistance across the joints before and after isothermal aging was measured by a homemade four-probe platform. Figure 2 is a graph showing the electrical resistance across the joint before and after isothermal aging. The electrical resistance goes smoothly across the joint and no sharp rising of electrical resistance appears at each interface of the joint except for the Ti-Al/SKD interface, indicating that the contact resistance at the HH/Ni/Ni-Sn/Ni/Ti-Al interfaces is very low before and after aging. This means the bonding layer not only forms good bonding with the connecting layer on both sides of HH and SKD but also has low electrical resistance before and after aging. As described above, the bonding layer and connecting layer formed intermetallic compounds (Ni_3_Sn_4_, Ni_3_Sn_2_, and Ni_3_Sn) and the HH/Ni interface changed into Ni_3_Sn and HH based compounds after joining and aging. These compounds have low electrical resistance. This is reason why the HH/Ni/Ni-Sn/Ni/Ti-Al interfaces have low contact resistance. A sharp rise of electrical resistance at the Ti-Al/SKD interface means that the contact resistance at this interface is much higher than those at other interfaces of the joint. This can attribute to the formation of IMCs at the interface of Ti-Al/SKD, especially the p type TiCoSb phase [31]. Contact resistivity at the Ti-Al/SKD interface was calculated as 9.4 μΩ·cm^2^ and 20.1 μΩ·cm^2^ before and after aging. The rising of the contact resistivity may result from significant increase of the amount of IMCs at the Ti-Al/SKD interface after aging [24,25,26].

## 4. Conclusions

- Segmented HH and SKD TE materials were successfully joined by a low temperature method and survived a 600 °C isothermal aging experiment.
- The transformation of Sn and Ni into Ni_3_Sn_4_ during the joining and following heat treatment and Ni_3_Sn_2_ and Ni_3_Sn during the isothermal aging is the reason why the joint was well bonded and could survive the high temperature isothermal aging.
- The contact resistance at the interface between the bonding layer and connecting layers of both HH and SKD is very low. The above results show that the low temperature joining process is effective to fabricate qualified segmented TE joints.
